# Adenoid cystic carcinoma of the external auditory canal with metastasis to the cerebellopontine angle mimicking vestibular schwannoma: a case report and literature review

**DOI:** 10.3389/fonc.2025.1680861

**Published:** 2026-01-12

**Authors:** Zhifei Guo, Jie He, Xiangyu Zhang, HaiYan Xu, Bing Zhao, Wei Cao

**Affiliations:** 1 Department of Neurosurgery, The Second Affiliated Hospital of Anhui Medical University, Cerebral Vascular Disease Research Centre, Anhui Medical University, Hefei, China; 2 Department of Otolaryngology, Head and Neck Surgery, The Second Affiliated Hospital of Anhui Medical University, Hefei, China

**Keywords:** adenoid cystic carcinoma, cerebellopontine angle, external auditory canal, metastasis, vestibular schwannoma

## Abstract

**Purpose/aim:**

Adenoid cystic carcinoma (ACC) of the external auditory canal (EAC) is a rare malignancy with a propensity for perineural invasion and distant metastasis. Its metastasis to the cerebellopontine angle (CPA) has not been previously documented. This case report presents a unique instance of CPA metastasis from EAC ACC, which mimicked vestibular schwannoma. We also review pertinent literature to enhance awareness of this atypical presentation.

**Case report:**

We present the case of a 62-year-old male with CPA metastatic ACC who experienced right facial paralysis and hearing loss for more than 5 years. The patient underwent a surgical resection of the ACC of the right EAC in the Otolaryngology, Head and Neck surgery department 10 years ago. The postoperative recovery was good, and symptoms of right facial paralysis began to appear in the fourth year after surgery. Reexamination via head Computed Tomography (CT) and Magnetic resonance imaging (MRI) revealed no local recurrence or distant metastasis. Over subsequent years, the patient’s symptoms of facial paralysis gradually worsened, and discomfort, such as eyelid ptosis, decreased vision, and hearing loss, occurred, MRI of the head revealed a cystic lesion located in the right CPA. On March 19, 2025, the patient underwent retrosigmoid craniotomy, the tumor was found to be located in the CPA, penetrating into the Internal auditory canal (IAC), with a solid and tough texture, abundant blood vessels, clear boundaries. Gross total resection was achieved. The patient was discharged on the 9th postoperative day with good recovery and without any new neurological dysfunction. Histopathological examination revealed a diagnosis of ACC. One month after surgery, the patient received adjuvant chemoradiation and was disease-free at 8-month follow-up after the second surgery.

**Conclusions:**

ACC of the EAC with intracranial extension into the CPA is an exceptionally rare clinical entity that may radiologically mimic vestibular schwannoma. To our knowledge, this is the first such case reported in the literature. From this rare case, neurosurgeons should understand that malignant pathologies must be considered when diagnosing CPA masses, particularly when atypical features such as perineural spread are present.

## 1 Introduction

Adenoid cystic carcinoma (ACC), also known as cylindroma, is a rare malignant tumor that frequently arises in the salivary glands, particularly the minor salivary glands of the palate, as well as major salivary glands, such as the submandibular and sublingual glands (1). ACC represents the most common malignant salivary gland tumor, accounting for approximately 10% of all salivary gland neoplasms and 30% of minor salivary gland tumors (2). Malignancies of the external auditory canal (EAC) are predominantly squamous cell carcinomas (80%), while ACC accounts for approximately 5% of such malignancies (3). The most common metastatic sites include the lungs, followed by the bone, liver, and kidney (4). Intracranial metastasis occurs in fewer than 5% of cases (5), typically involving the anterior or middle cranial fossa or cerebral convexity, and may radiographically resemble meningioma (5–7). As summarized in **Table 1**, documented cases of intracranial metastasis from EAC-derived ACC are limited, with only two prior reports in the English literature (8, 9). To our knowledge, metastasis to the cerebellopontine angle (CPA) has not been previously reported. Here, we present a case of ACC of the EAC metastasizing to the CPA a decade after initial resection, radiologically and clinically masquerading as a vestibular schwannoma. The aims of this study are to: 1) detail the clinical, radiological, and pathological findings of this novel presentation; 2) review the existing literature on EAC ACC and its metastatic patterns; and 3) highlight critical diagnostic and management considerations to prevent misdiagnosis and guide optimal patient care.

**Table 1:** Summary of cases of intracranial metastases from ACC of the EAC.

Authors, Year	Number cases	Age/sex	Symptoms	Metastatic location	Other distant metastases	Time to intracranial metastases	Previous treatment	Therapy following intracranial metastases
Conlin PA, et al 2002	1	38,Male	headaches	Parietal lobe	none	4 months	Surgery	Biopsy
Kuramitsu S, et al 2022	1	72,Male	Facial palsy & dysarthria	Frontal lobe	Lung, parotid lymph node	7 years	Surgery, chemotherapy	Surgery
Current case	1	62,Male	Facial palsy & Hearing loss	Right cerebellopontine angle	none	10 years	Surgery	Surgery, chemotherapy

## 2 Case presentation

**Figure 1:**
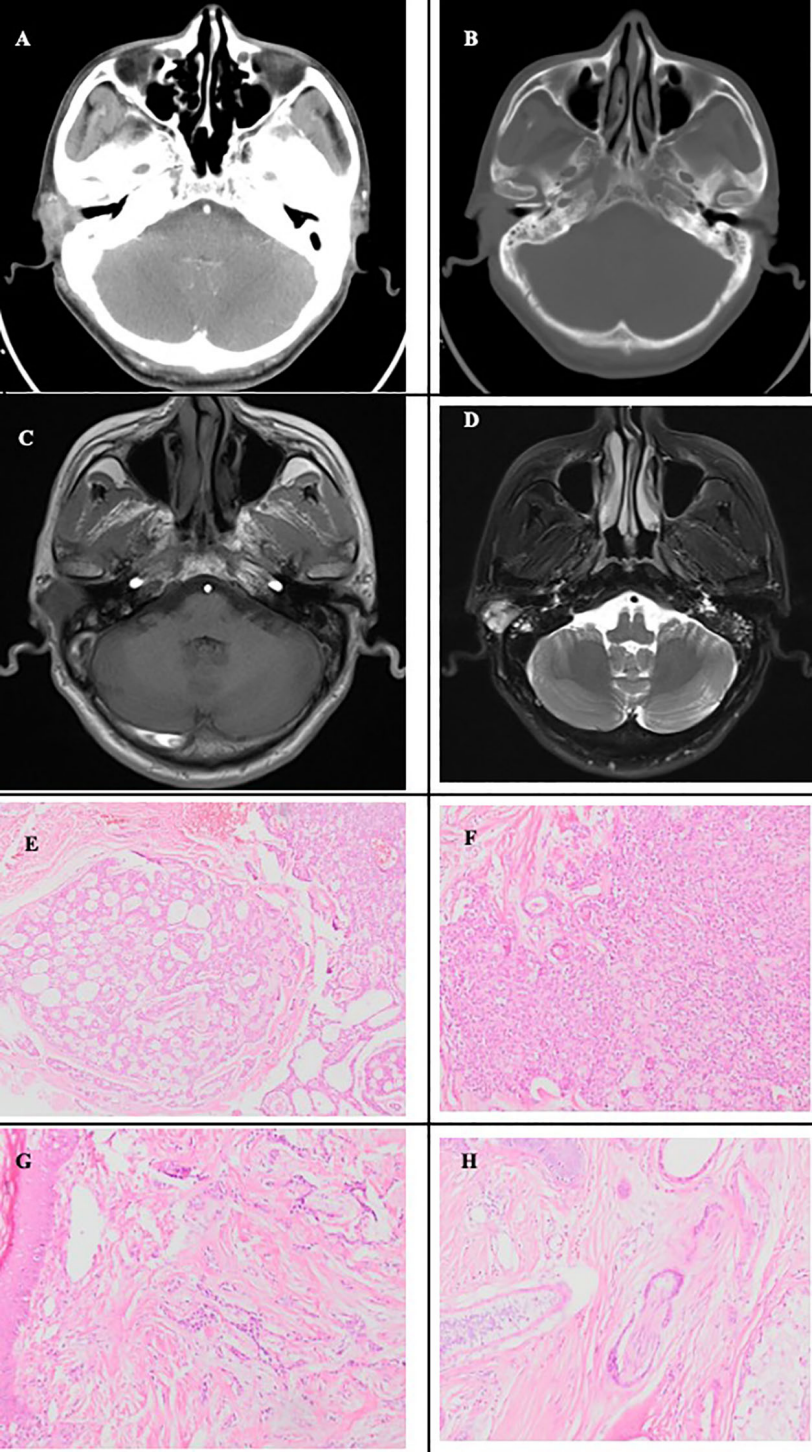
Preoperative imaging findings and histopathological features of the tumor of the first surgery. **(A)** Axial contrast-enhanced CT of the temporal bone demonstrates mild enhancement of the tumor. in the right EAC. **(B)** The bone window reveals no adjacent bone destruction. **(C, D)** Inner ear MRI shows a slightly hypointense T1 and hyperintense T2 signal mass in the right EAC: axial T1-weighted image **(C)**, axial T2-weighted image **(D)**. **(E)** Tumor cells arranged in a cribriform and microcystic pattern (HE staining, ×100). **(F)** Tumor cells forming solid nests (HE staining, ×200). **(G)** Tumor cells arranged in cord-like structures infiltrating the subdermal stroma (HE staining, ×200). **(H)** Perineural invasion in adenoid cystic carcinoma, with tumor cells encircling the nerve circumferentially (HE staining, ×200).

A 62-year-old male presented to Department of Otolaryngology, Head and Neck surgery at our hospital ten years ago with a 2-year history of a right EAC mass and 2 months of associated pain and bleeding. Physical examination revealed a nodular mass with a diameter of approximately 1.5 cm on the posterosuperior wall of the right EAC near the conchal cavity. The computed tomography (CT) plain scan and enhancement of the middle ear mastoid revealed a 1.2×1.5×1.7 cm^3^ irregular, poorly demarcated soft tissue mass with mild-to-moderate heterogeneous enhancement, without evident temporal bone destruction (**Figures 1A, B**). The magnetic resonance imaging (MRI) plain scan of the inner ear canal revealed a slightly longer T1 and T2 signal-occupying space in the right EAC (**Figures 1C, D**). There were no dilations in the bilateral internal auditory canal (IAC), and the auditory nerves were clearly displayed. The initial radiologic differential considerations included ceruminoma, benign soft tissue neoplasm, or less likely, a malignant tumor such as ACC. Subsequently, the patient underwent a puncture biopsy of an EAC mass, and pathology revealed ACC. On August 19, 2015, the patient underwent wide local excision including right EAC tumor and EAC resection, superficial parotidectomy, and modified radical mastoidectomy. Postoperative pathology confirmed ACC, cribriform subtype and the negative surgical margins of the EAC skin. No formal grading was provided in the initial report, but the morphology was consistent with a low-to-intermediate grade tumor (**Figures 1E–H**). The patient recovered well without complications such as facial paralysis and was discharged on time for follow-up.

**Figure 2:**
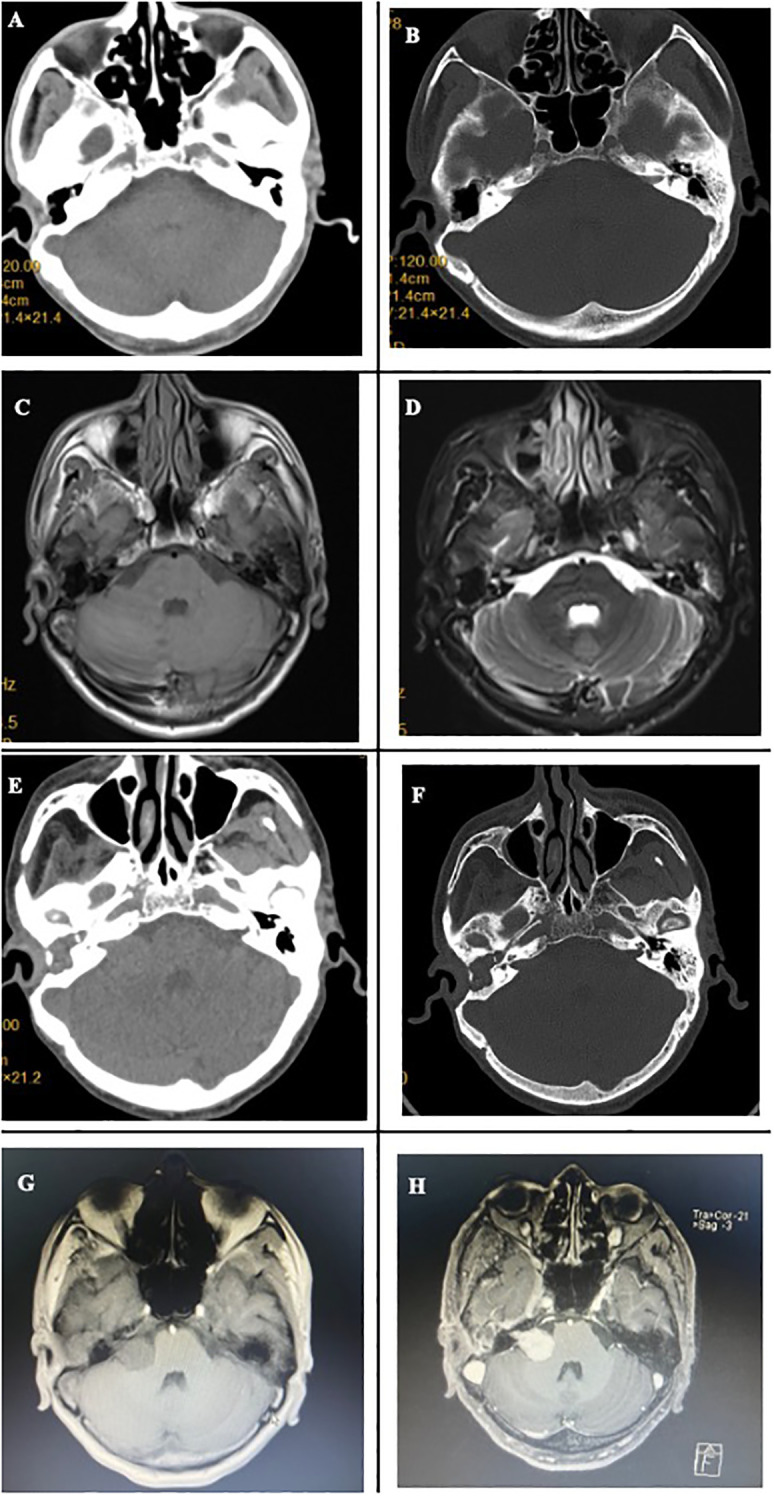
Imaging findings from 2019 and Preoperative imaging findings before the second surgery. **(A, B)** Noncontrast head CT and bone window show no tumor recurrence or metastasis in the intracranial region or right EAC. **(C, D)** Head MRI reveals no evidence of tumor recurrence or metastasis in the intracranial region or right EAC. **(E)** Noncontrast head CT shows an isodense mass in the right CPA. **(F)** CT bone window demonstrates significant widening of the right IAC. **(G)** Noncontrast head MRI reveals a slightly hypointense T1 signal mass in the right CPA. **(H)** Contrast-enhanced head MRI shows marked homogeneous enhancement of the right CPA mass, with tumor growth extending into the IAC.

In the fourth year after surgery, the patient presented with symptoms of right facial paralysis such as incomplete closure of the right eyelid and skewed corners of the mouth. Follow-up head CT and MRI plain scan showed no local recurrence or intracranial metastasis (**Figures 2A–D**). However, the facial nerve electromyogram indicated damage to the right lateral nerve, suggesting tumor recurrence and invasion of the facial nerve. The patient was advised to undergo chemoradiotherapy, but he declined adjuvant therapy.

**Figure 3:**
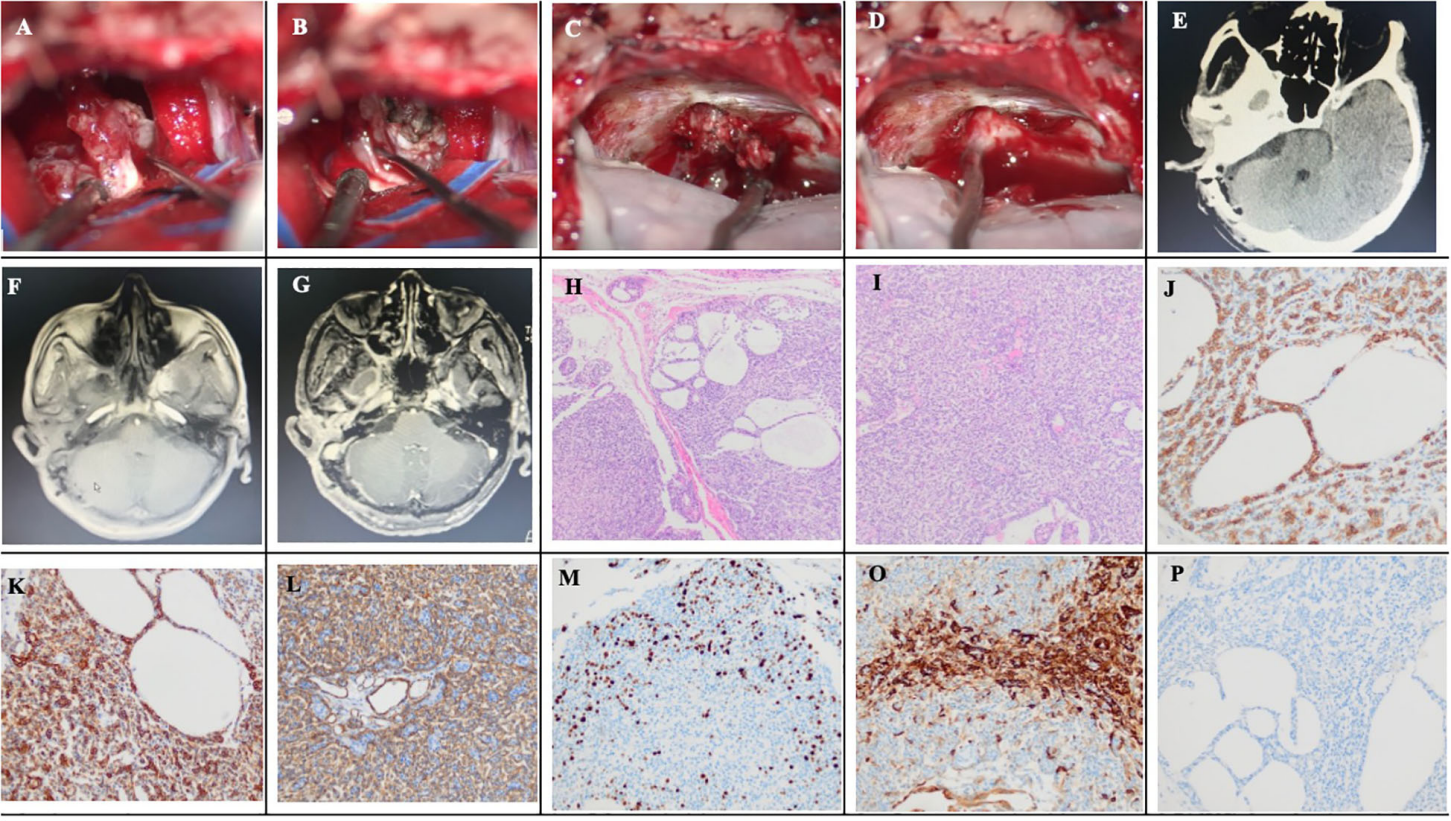
Intraoperative photographs, postoperative images and postoperative pathology of the second surgery. **(A)** The vestibulocochlear nerve is completely infiltrated and destroyed by the tumor. **(B)** The facial nerve is invaded and damaged by the tumor. **(C)** Tumor growth penetrating the IAC. **(D)** No identifiable vestibulocochlear or facial nerves near the IAC opening after tumor resection. **(E)** Postoperative Day 1 head CT shows no hemorrhage in the surgical area. **(F)** Postoperative noncontrast head MRI reveals no significant edema in the surgical region. **(G)** Postoperative contrast-enhanced head MRI confirms complete tumor resection. **(H)** Diffuse cribriform growth pattern of tumor cells (HE staining, ×100). **(I)** Diffuse solid growth pattern of tumor cells (HE staining, ×100). **(J)** CD117 positivity. **(K)** CK7 positivity. **(L)** SMA positivity. **(M)** Immunohistochemistry for Ki67. **(O)** Calponin positivity. **(P)** PR negativity.

Over subsequent years, the patient’s symptoms of facial paralysis gradually worsened, and discomfort such as eyelid ptosis, decreased vision, and hearing loss occurred. Therefore, on March 2, 2025, the patient returned to our outpatient department and underwent a head MRI scan, which revealed a space-occupying lesion in the right CPA (**Figures 2G, H**). The patient was admitted with the main complaint of “right facial paralysis, hearing loss for more than 5 years, and intracranial space-occupying lesion discovered for a week”. The physical examination at admission revealed the following: clear consciousness, drooping right eyelid, dilated right pupil, disappearance of the light reflex, decreased visual acuity in the right eye, left pupil diameter of 3 mm, sensitivity to the light reflex, hearing loss in the right ear, skewed right corner of the mouth, disappearance of forehead wrinkles, and normal and symmetrical facial sensation on both sides. Head MRI now revealed a 1.5×2×2 cm³ homogeneously enhancing space-occupying lesion in the right CPA. The lesion showed low T1 and high T2 signal intensity, extended into the widened IAC, and had an indistinct boundary with the auditory nerve (**Figures 2G, H**). A thin-layer CT of the mastoid process revealed enlargement of the right IAC (**Figures 2E, F**). The preoperative diagnosis favored vestibular schwannoma, although metastatic disease was a consideration. On March 19, 2025, the patient underwent retrosigmoid craniotomy, and the tumor was found to be located in the CPA, with a solid and tough texture, abundant blood vessels, clear boundaries, and no obvious adhesion to the brainstem and the cranial nerves V and IX-XII. The facial nerve was infiltrated and tumorified by the tumor, and only a small amount of facial nerve stump could be seen at the root of the facial nerve (**Figures 3A, B**), with no electrophysiological activity. The tumor penetrated into the IAC (**Figure 3C**). First, the tumor was excised piece by piece. Then, the posterior wall of the IAC was ground open. There was no electrical activity of the facial nerve in the IAC, and the facial nerve had completely disappeared. Gross total resection was achieved under a microscope (**Figure 3D**). A postoperative head CT scan revealed no abnormalities such as bleeding in the surgical area (**Figure 3E**). Postoperative gadolinium-enhanced MRI revealed total tumor resection (**Figures 3F, G**). The patient recovered well and was discharged on the 9th day without any new neurological dysfunction. Postoperative pathology showed that the tumor cells were round or oval in shape, with active cell proliferation and visible mitotic figures, and were arranged in a solid, nest like, and sieve-like pattern. Immunohistochemistry indicated that Ki67 index was approximately 40%, and CD117 and CK7 were positive (**Figures 3H–P**). Based on the above pathological results, the patient was diagnosed with intracranial metastatic ACC of the EAC. One month after surgery, Given the diagnosis of intracranial metastatic ACC with extensive perineural invasion (as evidenced by facial nerve destruction) and a high Ki-67 index, the multidisciplinary tumor board recommended adjuvant chemoradiotherapy to the postoperative bed and at-risk neural pathways to mitigate the high risk of local recurrence. One month after surgery, the patient received adjuvant chemoradiation, adjuvant radiotherapy details: the patient underwent intensity-modulated radiation therapy (IMRT). The clinical target volume included the right CPA, middle skull base, temporal bone, and the right parotid region. A total dose of 60 Gy in 30 fractions was delivered to the high-risk postoperative bed. The patient tolerated the treatment well without significant acute toxicity and was disease-free at 8-month follow-up after the second surgery.“

## 3 Discussion

ACC can occur at any age (1–90 years old), with an average age of onset of 50 years old. It is more common in middle-aged or older individuals and is slightly more common in females (2, 10). This tumor infiltrates between tissues, so the clinically visible mass is often small, while in reality, the tumor has a wide range of involvement. Tumors sometimes have intracranial invasion, similar to meningiomas in terms of clinical and imaging aspects, and some cases have special clinical manifestations such as cavernous sinus syndrome (6, 7). The most common clinical symptoms of EAC ACC are ear pain and EAC mass (11, 12). During the patient’s initial examination, a small nodule was observed on the posterior wall of the EAC near the conchal cavity, accompanied by ear pain, which is consistent with the common clinical manifestations of ACC in the EAC. EAC ACC is prone to invade the parotid gland, with a reported invasion rate of 35%, and parotid invasion is associated with poor survival rates without distant metastasis (13). Because there was no invasion of the parotid gland, the prognosis of this patient is relatively good. The local recurrence rate of EAC ACC is approximately 30%, with an average recurrence time of approximately 8 years (3). The distant metastasis rate is approximately 20–30% (13–15), with an average occurrence time of 34.8 months (10). The most common site of distant metastasis is the lungs (11), accounting for 97% of all cases (4). Distant metastasis to the central nervous system is rare. Currently, there are only 3 reported cases of intracranial metastasis (8, 9, 11) and 1 case of intraspinal metastasis (16). ACC is highly invasive, and approximately 60% (8% to 98%) and 40% of ACC patients experience nerve invasion and hematogenous metastasis, respectively (17, 18). Literature summary on recurrence and metastasis rates of EAC ACC were summarized in **Table 2**.

**Table 2:** Literature summary on EAC ACC.

Clinical aspect	Reported incidence/data
The incidence of parotid gland invasion in EAC ACC	35%
Frequency among EAC malignancies	Approx.5%
Local recurrence rate	Approx. 30% (avg. time: ~8 yrs)
Distant metastasis rate	20-30% (lungs most common)
Intracranial metastasis rate	<5% (anterior/middle fossa)
5-year survival (with clear margins)	89%

ACC, Adenoid cystic carcinoma; EAC, external auditory canal.

The common tumors in the intracranial CPA include vestibular schwannoma, meningioma, and epidermoid cyst, among which vestibular schwannoma is the most common. Vestibular schwannoma presents as hearing loss, and imaging reveals a space-occupying lesion in the CPA. The tumor invades and grows in the IAC, and the IAC enlarges. In this case, the radiological features of the CPA lesion, including IAC enlargement and homogeneous enhancement, closely mimicked a vestibular schwannoma. However, a CPA schwannoma of this size rarely causes facial paralysis, even if it originates from the facial nerve itself. These conflicting features made a definitive non-surgical diagnosis challenging, surgical intervention was necessary for pathological confirmation. It is obviously different from acoustic neuroma according to intraoperative observations. Acoustic neuroma generally presents as yellow fat-like changes, accompanied by cystic changes, and it is usually attached to the brainstem and cerebellum. Although the facial nerve is thin under pressure, it is located outside the facial mask structure on the surface of the tumor, and can be identified and separated. In this case, the tumor was completely fused with the facial and auditory nerves. The tumor texture is tougher than that of acoustic neuroma, and the blood supply is less abundant than that of acoustic neuroma. The boundary between the brain stem and cerebellum is gray and clear, without obvious adhesion. These are characteristics that acoustic neuromas do not have. The pathological feature of ACC is CD117/CK7 positivity, whereas the pathological feature of acoustic neuroma is S-100 protein positivity (19). In addition, the Ki67 index of this patient’s tumor was as high as 40%, while the Ki67 index of acoustic neuroma is generally approximately 5%. Therefore, immunohistochemistry does not match the presentation of acoustic neuroma.

The main treatment for ACC is mainly surgical resection, which should expand the resection range, especially for the first resection, and requires intraoperative frozen section examination to determine whether the resection margin is negative. Gu et al. conducted a statistical analysis of the prognosis of 43 patients with EAC ACC who underwent surgery, and the results revealed that the 5-year survival rates of 19 patients with clear surgical margins and 24 patients with positive surgical margins were 89% and 54%, respectively (12). Temporal bone resection is strongly recommended to improve the local control rate and the reduce recurrence rate (14). This patient underwent tumor resection, EAC resection, superficial parotid gland resection, and modified radical mastectomy for the first time, so there was no significant local recurrence 10 years after surgery. However, due to the tumor’s tendency to invade nerves and metastasize, distant intracranial metastasis still occurred. For patients in the middle and late stages of ACC, chemotherapy can delay tumor metastasis and spread. The most commonly used chemotherapy regimens are platinum-based monotherapy and combination therapy with other drugs (17). Radiation therapy is suitable for patients with nonnegative margins or late-stage metastases (12). While achieving negative surgical margins is a primary goal and a strong positive prognostic factor, the decision for adjuvant radiotherapy must be individualized based on a comprehensive risk assessment. For patients with truly low-risk features (e.g., early T-stage, margin-negative resection without perineural invasion or other adverse factors), observation may be considered. However, adjuvant radiotherapy is frequently recommended in the presence of high-risk features such as positive or close margins, extensive perineural invasion (especially named nerve involvement), advanced T-stage, recurrent disease, or skull base involvement, where it has been shown to improve local and regional control (20, 21). The impact of adjuvant radiotherapy on overall survival in ACC remains less clearly defined due to the disease’s propensity for late distant metastasis. Conventional radiotherapy is also insensitive to tumors, but in recent years, carbon ion radiotherapy (CIRT) has emerged as a new radiation therapy technique. Targeted therapy and immunotherapy have also shown certain efficacy and feasibility. In summary, although there is currently no established or highly effective chemoradiotherapy (CRT) regimen specifically for ACC, previous studies have reported that CRT can be beneficial in controlling disease progression for patients with recurrent, metastatic, or incompletely resected ACC (11, 22, 23).

The prognosis of ACC is closely related to the tissue type of the tumor, and poor survival predictors include advanced T stage, lymph node positivity, solid tumors, distant metastasis, and positive surgical margins (10). The WHO classifies ACC into tubular-type (grade I), sieve-type (adenoid type, grade II), and solid-type (grade III), with sieve-type being the most common and solid-type being the least common (24). Compared with tubular- and sieve-type, solid-type cases have a poorer prognosis and higher recurrence and metastasis rates (25). The pathology of this patient suggests that the tumor belongs to the sieve-type, and that the prognosis is relatively good. The T stage of tumors has a significant impact on patient prognosis. The 5-year survival rates of patients with T stage 1 to 4 are 85%, 67%, 67%, and 30%, respectively (12). The involvement of the dura mater, facial nerve, sigmoid sinus, deep lobe of the parotid gland, and parapharyngeal space is significantly correlated with poor prognosis of in EAC ACC patients (26). The median survival time after distant metastasis is 13 months, and the prognosis of simple lung metastasis is better than that of other internal organ or bone metastases (15).

On the basis of this case analysis combined with the literature, we can draw the following lessons and experience summary: 1) Once a patient has a history of ACC and presents with symptoms of cranial nerve damage, whether or not a space occupying lesion is found in the skull, it should be highly suspected that the ACC has distant intracranial metastasis and invasion of nerves, even if the morphology and location of the intracranial tumor are typical of primary intracranial tumors such as meningiomas or acoustic neuromas. 2) The degree of initial resection is crucial for the prognosis of EAC ACC. It is recommended to enlarge the resection range as much as possible to ensure a negative margin, and to remove the parotid gland to minimize local recurrence and improve patient prognosis. 3) Given the high risk of late recurrence and metastasis, long-term (often spanning decades) or even lifelong follow-up is imperative. Once symptoms related to cranial nerves appear, even if there are no obvious metastatic lesions, timely radiotherapy and chemotherapy are needed to curb tumor invasion and growth.

## 4 Conclusion

ACC of the EAC with intracranial extension into the CPA is an exceptionally rare clinical entity that may radiologically mimic vestibular schwannoma. This case highlights a critical clinical implication: in any patient with a history of ACC, even a remote one, who presents with new cranial neuropathies or a CPA mass, metastatic disease must be a primary consideration, regardless of how typical the imaging appears for a benign lesion like vestibular schwannoma. This high index of suspicion is paramount for timely diagnosis and appropriate management. Given EAC ACC’s propensity for perineural invasion, delayed recurrence, and distant metastases, clear margins remain the cornerstone of treatment, while adjuvant radiotherapy may improve local control. Long-term follow-up is imperative due to the indolent yet aggressive nature of these tumors. Multidisciplinary collaboration among otologists, neurosurgeons, and oncologists is critical to optimize outcomes for these complex cases. The primary limitation of this study is its nature as a single case report. Its novelty lies in documenting a previously unreported metastatic site for this rare tumor. Future efforts should focus on establishing long-term registries for rare tumors like EAC ACC and exploring molecular biomarkers to better predict metastatic behavior and guide targeted therapies.

## Data Availability

The original contributions presented in the study are included in the article/supplementary material. Further inquiries can be directed to the corresponding authors.
